# Cashew Nut Shell Liquid (CNSL) (Feeds, Fertilizers, etc.)

**DOI:** 10.14252/foodsafetyfscj.D-25-00020

**Published:** 2025-06-28

**Authors:** Food Safety Commission of Japan

## Abstract

Food Safety Commission of Japan (FSCJ) conducted a risk assessment of a feed additive, cashew nut shell liquid (CNSL), referring to the submitted documents for feed additive designation. FSCJ assessed anacardic acid in May, 2024 as an active substance of this formulation, concluded that this substance would not have a negative effect on human health as long as normally used as a feed additive. This additive was administered to cattle after mixing it into their feed in residue study. CNSL was, however, not detected in either tissues or milk. FSCJ thus recognized that humans would not have detectable amounts of CNSL from food through the feed additive. FSCJ concluded that negligible effects on human health as long as used ordinally as a feed additive.

## Conclusion in Brief

Food Safety Commission of Japan (FSCJ) conducted a risk assessment of a feed additive, cashew nut shell liquid (CNSL), referring to the submitted documents for feed additive designation

CNSL is a feed additive to reduce methane emissions from cattle belching. This additive is applicable up to 0.1%.

FSCJ assessed anacardic acid in May, 2024 as an active substance of this formulation, concluded that this substance would not have a negative effect on human health as long as normally used as a feed additive.

FSCJ judged that the excipients in this feed additive would be negligible effects on human health, considering their usage and previous assessments, in the cases of taking this feed additive as an ingredient.

This additive was administered to cattle after mixing it into their feed in residue study. CNSL was, however, not detected in either tissues or milk. FSCJ thus recognized that humans would not have detectable amounts of CNSL from food through the feed additive.

From the results of safety and feeding tests in cattle, FSCJ judged no safety concern for the cattle administered this feed additive with the recommended levels.

Given the above, FSCJ concluded that negligible effects on human health as long as used ordinally as a feed additive.
